# Predictive Factors Associated with Complications after Laparoscopic Distal Pancreatectomy

**DOI:** 10.3390/jcm9092766

**Published:** 2020-08-26

**Authors:** Ki Byung Song, Sarang Hong, Hwa Jung Kim, Yejong Park, Jaewoo Kwon, Woohyung Lee, Eunsung Jun, Jae Hoon Lee, Dae Wook Hwang, Song Cheol Kim

**Affiliations:** 1 Division of Hepatobiliary and Pancreatic Surgery, Department of Surgery, Asan Medical Center, University of Ulsan College of Medicine, 88 Olympic-ro 43-gil, Songpa-gu, Seoul 05505, Korea; mtsong21c@naver.com (K.B.S.); 8thofnovember@hanmail.net (S.H.); blackpig856@gmail.com (Y.P.); skunlvup@naver.com (J.K.); ywhnet@gmail.com (W.L.); eunsungjun@amc.seoul.kr (E.J.); hbpsurgeon@gmail.com (J.H.L.); drdwhwang@gmail.com (D.W.H.); 2 Department of Clinical Epidemiology and Biostatistics, Asan Medical Center, University of Ulsan College of Medicine, 88 Olympic-ro 43-gil, Songpa-gu, Seoul 05505, Korea; rsvp@amc.seoul.kr

**Keywords:** laparoscopic distal pancreatectomy, risk factors, complication, surgery-related factors, postoperative pancreatic fistula

## Abstract

Although laparoscopic distal pancreatectomy (LDP) has become more popular, the postoperative complication rate remains high. We sought to identify the risk factors for post-LDP complications. We examined 1227 patients who underwent LDP between March 2005 and December 2015 at a single large-volume center. We used logistic regression for the analysis. The overall (13.2%) and major (3.3%) complication rates were determined. Postoperative pancreatic fistula was the most frequent complication, and 58 patients (4.7%) had clinically significant (grade B) pancreatic fistulas. No 90-day mortality was recorded. Long operative time (≥200 min), large estimated blood loss (≥320 mL), LDP performed by an inexperienced surgeon (<50 cases), and concomitant splenectomy were identified as risk factors for overall complications using a logistic regression model. For major complications, male sex (*p* = 0.020), long operative time (*p* = 0.005), and LDP performed by an inexperienced surgeon (*p* = 0.026) were significant predictive factors. Using logistic regression analysis, surgery-related factors, including long operative time and LDP performed by an inexperienced surgeon, were correlated with overall and major complications of LDP. As LDP is a technically challenging procedure, surgery-related variables emerged as the main risk factors for postoperative complications. Appropriate patient selection and sufficient surgeon experience may be essential to reduce the complications of LDP.

## 1. Introduction

Laparoscopic distal pancreatectomy (LDP) has gained an increased popularity in recent years, and numerous reports have been published on the advantages of LDP as a treatment option for patients with left-sided pancreatic tumors. Compared with open surgery, the laparoscopic approach has been reported to be safe and associated with less blood loss, more rapid recovery, shorter hospital stay, and comparable oncologic outcomes [1,2,3,4]. However, postoperative complications are the main causes of increased hospitalization time, resource use, and decreased quality of life. The most frequent complications after distal pancreatectomy are postoperative pancreatic fistula (POPF) and complicated fluid collection [5]; however, the determinants of post-LDP complications are poorly defined. It is essential to validate the risk factors for surgical morbidity using a large patient cohort. Therefore, in this study, we used a large single-center data set to analyze the risk factors for post-LDP complications and to determine the effects of patient characteristics, surgery-related factors, and pathologic findings on postoperative complications in patients undergoing LDP.

## 2. Materials and Methods

### 2.1. Patients

We retrospectively reviewed the data of all patients who underwent LDP performed by five pancreatic surgeons from March 2005 to December 2015 at a single large-volume center, after receiving institutional review board approval (No. 2016-0612). The collected data included patient demographics, operative variables, postoperative outcomes, and pathologic findings. Patients who underwent neoadjuvant therapy were excluded and we identified final 1227 patients who underwent LDP between 1 March 2005 and 31 December 2015, from the electronic database of the center. We evaluated the patient records to assess the risk factors for perioperative complications. The patients were followed up with abdominal computed tomography (CT) scans and blood tests (including tumor markers) every 3 months for the first 2 years after LDP and every 3–6 months thereafter.

### 2.2. Definitions

Operative time was calculated as the time from skin incision to skin closure. Estimated blood loss was calculated as previously described [6]. Surgical and medical complications were graded according to the Clavien–Dindo system. Grade I and II complications were considered minor morbidity, whereas major morbidity was defined as being complications higher than grade III [7]. We assigned the highest-grade complication experienced by each patient as the final overall complication grade. Placement of drains after LDP was performed as a routine procedure at our hospital. We routinely checked the Jackson-Pratt drain amylase/lipase level on postoperative day (POD) #1 and #3 and removed drain unless drain amylase level was high. We routinely performed postoperative CT on POD #3. The grade of POPF and the status of postoperative fluid collection were determined on the basis of the evaluations. POPF was defined according to the consensus definitions of the 2016 International Study Group of Pancreas Surgery [8]. When postoperative fluid collection was combined with leukocytosis and elevated C-reactive protein and treated with antibiotics, it was classified as a grade II complication. Pathologic data included tumor size, histologic grade, and resection margin status.

### 2.3. Operative Technique

As previously described [3,4], to safely transect the pancreas during laparoscopy, we used endoscopic linear staplers of various heights, depending on the thickness or hardness of the pancreas. Two endoscopic stapler cartridges, Echelon Endopath (Ethicon Endosurgery, Cincinnati, OH, USA) and EndoGIA™ 60-mm Reload with Tri-Staple™ Technology (Covidien Medtronic, Plymouth, MN, USA), included (i) a regular-height cartridge (3.8-mm gold Echelon Endopath or 3- to 4-mm purple EndoGIA™ 60-mm Reload with Tri-Staple™ Technology), (ii) a long-height cartridge (4.1-mm green Echelon Endopath or 4- to 5-mm black EndoGIA™ 60-mm Reload with Tri-Staple™ Technology), and (iii) a short-height cartridge (3.5-mm blue Echelon Endopath).

In most cases, after transecting the pancreas, the pancreatic remnant was covered with a fibrinogen-coated and thrombin-coated collagen sponge (TachoComb or TachoSil; Nycomed GmbH, Konstanz, Germany) or a polyglycolic acid felt (Neoveil^®^) and fibrin sealant (Tissucol; Baxter GmbH, Unterschleissheim, Germany).

### 2.4. Statistical Analysis

We used SPSS version 24.0 for all statistical analyses (SPSS Inc., Chicago, IL, USA). Multiple logistic regression models were used to determine the patient characteristics, operative factors, and pathologic findings that were potentially related to overall and major complications. All continuous data fields were made dichotomous around the mean value, or according to accepted cutoff points in the literature. The results of multivariate analyses are expressed as odds ratios (=Exp(B)) with corresponding 95% confidence intervals around the means of variables that predicted an increased risk of major complications (*p* < 0.05).

### 2.5. Evaluation Factors

We performed multivariate analyses using the candidate variables that were introduced as risk factors for complications after distal pancreatectomy in previous studies, as discussed below [9,10,11,12,13,14,15,16,17,18,19,20].

#### 2.5.1. Patient-Specific Factors

Age (<70 vs. ≥70 years), sex (female vs. male), and preoperative American Society of Anesthesiologists (ASA) scores were recorded. Serum albumin levels (<3.5 vs. ≥3.5 g/dL) were determined during routine preoperative evaluations. Preoperative body mass index ((BMI), normal vs. overweight vs. obese according to the World Health Organization classification) was calculated as body weight in kilograms divided by height in meters squared.

#### 2.5.2. Surgery-Related Factors

Data on operative time (<200 vs. ≥200 min) and estimated blood loss (<320 vs. ≥320 mL) were collected, and the presence of a concomitant splenectomy or multivisceral resection was evaluated. Multivisceral resection was defined as a concomitant resection of one of the following: stomach, colon, small bowel, or kidney. Surgeons who had performed <50 LDP procedures were classified as inexperienced surgeons, whereas those who had performed ≥50 LDP procedures were considered experienced surgeons. Every surgeon had sufficient experience of open DP, laparoscopic cholecystectomy, and laparoscopic splenectomy before beginning LDP.

#### 2.5.3. Pathologic Factors

The presence of a malignant disease was verified through a review of the original histopathologic evaluation. The extent of pancreatic resection (<12 vs. ≥12 cm) was determined according to the length of the gross pancreatic specimen. Pancreatic tumors were divided into malignant and benign.

## 3. Results

### 3.1. Patients’ Demographics and Tumor Factors

The characteristics of all patients and tumors are shown in Table 1. Of the 1227 patients included, 498 were men and 729 were women. The mean age at the time of surgery was 52.9 years (standard deviation (SD), 14 years). The mean tumor size was 3.6 cm (SD, 2.3 cm). Of the lesions, 978 (79.7%) were benign or borderline malignant and 249 (20.3%) were malignant at the time of resection. The most common indication for resection was an intraductal papillary mucinous neoplasm (n = 238, 19.4%), followed by pancreatic cancer (n = 218, 17.8%) and a solid pseudopapillary neoplasm (n = 178, 14.5%).

### 3.2. Surgical Technique and Operative Details

The operative data are summarized in Table 2. Of the 1227 patients who underwent LDP, 573 (46.7%) underwent spleen-preserving-LDP (SP-LDP): 417 with main splenic vessel preservation and 156 with preservation of short gastric and gastroepiploic vessels (Warshaw technique). A total of 176 patients underwent LDP combined with other operations. The mean operative time was 197 min (SD, 64.7 min). The mean length of hospital stay (LOS) was 9 days (SD, 6.5 days). On average, a normal diet was resumed 2.1 days (SD, 1.3 days) after the surgery.

### 3.3. In-Hospital Complications

In-hospital complications graded using the Clavien–Dindo classification are summarized in Table 3. The overall complication rate was 13.2%. Most patients experienced either no (n = 1065, 86.8%) or minor (n = 121, 9.9%) Clavien–Dindo I/II events. Major morbidity (Clavien–Dindo III/IV/V) occurred in 41 patients (3.4%), of which POPF was most frequent, and 58 patients (4.7%) had clinically significant (grade B) fistulas. A total of 32 patients had grade B POPF and required endoscopic ultrasound-guided or percutaneous drainage. The most common life-threatening postoperative complication was bleeding (n = 5, 0.4%). One case of bleeding was treated angiographically by embolization and four were treated surgically. The postoperative complications necessitated reoperation in seven patients (0.6%): four patients for postoperative bleeding, two for mechanical ileus, and one for common bile duct injury. No 90-day mortality was recorded.

### 3.4. Logistic Regression Analysis of Risk Factors for Post-LDP Complications

To identify the risk factors for perioperative complications after LDP, we retrospectively analyzed the clinicopathologic and operative variables of 1227 patients who underwent LDP. On the basis of a logistic regression model, long operative time (≥200 min), large estimated blood loss (≥320 mL), LDP performed by an inexperienced surgeon (<50 cases), and concomitant splenectomy were identified as significant factors for overall complications. For major complications, male sex (*p* = 0.020), long operative time (*p* = 0.005), and LDP performed by an inexperienced surgeon (*p* = 0.026) were significant predictive factors (Table 4).

## 4. Discussion

LDP is an alternative to open distal pancreatectomy (ODP) and is performed for benign and malignant tumors in the pancreatic body and tail. The perioperative and long-term oncologic outcomes of LDP, as currently practiced, are comparable to those of ODP [1,21,22,23,24]. Several reports have attempted to identify the risk factors for the development of complications, including POPF, after ODP [10,11,12,14,25]. However, few data are currently available to clarify the risk factors associated with post-LDP complications in a larger cohort.

From March 2005 to December 2015, a total of 1227 LDP procedures were performed by five hepatobiliary pancreatic surgeons at a single large-volume center. After LDP, 162 patients (13.2%) developed postoperative complications, including 58 patients (4.7%) with clinically relevant POPF. Of these, 41 complications (3.3%) were considered major complications according to the Clavien–Dindo classification.

In this study, the potential risk factors for overall and major complications were identified using binary logistic regression analyses. Previous reports have indicated that increased operative time is independently associated with an increased risk of complications after laparoscopic surgery [26,27]. On multivariate analysis in the present study, longer operative time (≥200 min) was associated with postoperative complications after LDP. Although the exact mechanism of the association between complications and long operative time is not fully understood, a prolonged operative duration can be attributable to various time-related factors such as prolonged microbial exposure and increased tissue retraction, leading to tissue ischemia and necrosis. In addition, longer operative times can be indicative of more complex or difficult surgeries, which would expectedly yield higher rates of complications. A large volume of blood loss was also identified as a risk factor for overall complications, using a logistic regression model. The negative impacts of prolonged surgery and increased blood loss are predictable because they likely serve as surrogate markers of technical difficulties that surgeons experience. Surgeons and surgical staff can positively influence patient outcomes by improving their technical skills and operative efficiency to reduce the operative time and blood loss. Surgeon-specific factors are known to affect postoperative outcomes. Surgeons in high-volume centers can achieve better outcomes than their counterparts in low-volume centers [28,29]. LDP is considered an advanced surgical approach because of its associated technical difficulties and prolonged learning curve. Even surgeons with extensive experience in performing ODP experience a high morbidity rate during their first few LDP procedures. We assessed the relationship between the surgeon’s experience and perioperative outcomes. In the present study, an inexperienced surgeon was defined as a surgeon who had performed <50 LDP procedures, whereas an experienced surgeon was defined as a surgeon who had performed ≥50 LDP procedures. We found that inexperienced surgeons had a 2.3-fold higher risk of complications than experienced surgeons. The operator learning curve therefore seems important for preventing post-LDP complications. In conclusion, surgery-related factors such as long operative time (≥200 min), large volume of estimated blood loss (≥320 mL), and LDP performed by an inexperienced surgeon (<50 cases) were major risk factors for postoperative complications. To reduce the risk of complications, surgeons should use expeditious surgical techniques and optimize patient selection to avoid immoderate surgery for LDP.

In the present study, SP-LDP was performed in 573 patients (46.7%). Spleen preservation during LDP remains controversial based on the various indications for pancreatic resection. Preservation of the spleen during distal pancreatectomy for ductal adenocarcinoma of the pancreas is formally contraindicated because it is an incomplete oncologic surgery, especially when lymph nodes along the splenic vessels and hilum are removed. However, as the role of the spleen has become better understood over the recent decades, SP-LDP has emerged as a first-choice operation for benign or low-grade malignant tumors located within the body or tail of the pancreas. Several studies showed higher complication rates after distal pancreatectomy with concomitant splenectomy [30,31,32]. In the present study, concomitant splenectomy was associated with more post-LDP complications. This may be due to devascularization of the pancreatic remnant in patients with concomitant splenectomy and delayed wound healing in the pancreatic stump. SP-LDP is considered an acceptable procedure for benign pancreatic tumors. However, some surgeons may not agree with the value of retaining the spleen in adult patients. In addition, SP-LDP (especially the Kimura technique) is a more tedious procedure with a greater chance of adjacent vascular injury and bleeding. Considering the technical demands of SP-LDP compared with conventional surgery, some may argue against efforts to preserve the spleen in adults during distal pancreatectomy. Therefore, more studies are needed to identify how splenectomy affects postoperative complications after LDP.

Obesity increases the risk inherent to major gastrointestinal surgeries [33] and is a confounding risk factor for surgical morbidity in patients undergoing pancreatic resection [15,34]. More precisely, visceral obesity is a major factor that increases postoperative complications [35,36]. Difficult manipulation, poor laparoscopic view, prolonged operative time, and the presence of a fatty or friable pancreas are related to visceral obesity and can be risk factors for postoperative complications [37,38]. In the present study, high BMI did not emerge as a risk factor for post-LDP complications. However, male patients had a 2.1-fold higher risk of developing major complications after LDP than female patients. Men have more intra-abdominal visceral adipose tissue than women [39]. Similar to our study, a past investigation found that male sex was a significant predictor of increased operative time, length of stay, transfusions, blood loss, and postoperative surgical-site infections [9]. Male sex is a risk factor for morbidity after LDP owing to the increased likelihood of visceral obesity and the technical difficulty associated with operating on patients with visceral obesity. However, to reach a clearer conclusion, visceral obesity should be precisely measured and its relationship with the likelihood of future complications should be evaluated.

Several techniques are used to close the pancreatic stump, and numerous techniques have been developed with the goal of reducing the incidence of POPF; however, the superiority of any particular closure technique has not been convincingly demonstrated [18,40]. In our center, transection and occlusion of the pancreas are achieved using an endoscopic linear stapler in most cases. We select the optimal cartridge height and use the parenchymal flattening technique [41] to reduce pancreatic damage during LDP. The mechanical jaw of the stapler should be closed gently, and the pancreas should be cut slowly to avoid causing any tissue damage. Furthermore, the stapler should not be released immediately after firing to prevent immediate bleeding from the pancreatic stump. A friable pancreas can be associated with POPF [26]. Some published papers have discussed the relationship between the thickness of the pancreatic stump and POPF [42]. A thick pancreas is more likely to be crushed during the procedure, causing the parenchyma to tear when it is compressed by the stapler. This might be a major risk factor for POPF. We selected the height of the cartridge of the stapler according to the thickness on the cutting line, as assessed intraoperatively. Regular-height cartridges were used in 984 patients (80.2%); long-height cartridges and suture reinforcement were also used if the pancreas was too thick, as was the case in 193 patients (15.7%); and a short-height cartridge was used in 50 patients (4.1%). In the present study, we did not include the texture and thickness for analysis. Because of the retrospective nature of the study, most data were incomplete for the evaluation of the texture and thickness of the pancreas. Therefore, this study has the limitation of not considering the thickness and texture of the pancreas as potential risk factors.

Theoretically, pathophysiologic events that occur secondary to an advanced malignancy, such as malnutrition and hypoalbuminemia, may contribute to impaired tissue healing, thereby increasing the likelihood of pancreatic fistulas; however, our data did not show a substantial impact of these factors on postoperative complications. We also assessed the correlation between postoperative complications and resected pancreatic length, and found no clear association.

In this study, we demonstrated a reduced rate of POPF compared to previous studies. This encouraging result seems to be due to the following four reasons. First, we used a slow parenchymal flattening technique when compressing and transecting the pancreas using an endoscopic linear stapler. This technique has been reported to reduce pancreatic damage [41]. Second, we usually remove abdominal drain on POD #3 unless drain amylase level is high. Several reports have proved that early removal of drain reduces POPF [43,44,45]. Third, operators in this study are highly experienced. As shown in the result, the more experienced operators can perform LDP with better postoperative outcomes. Finally, Asian people generally have a lower BMI compared to Western people, and thus soft and fatty pancreatic parenchyma, which are risk factors of POPF, are less common in Asian people than in Western people.

## 5. Conclusions

In conclusion, LDP is a technically feasible procedure that can be performed with acceptable operative morbidity and mortality. However, potential risk factors for overall and major surgical morbidities were identified on multivariate logistic regression analyses, including surgery-related factors such as long operative time and LDP performed by an inexperienced surgeon. As LDP is a technically challenging procedure, these surgery-related factors seem to increase the risk of postoperative complications. Appropriate patient selection and sufficient surgeon experience may be essential to reduce the complications of LDP.

## Figures and Tables

**Table 1:** Patient demographics and pathologic data.

Characteristic		
Patients, n	1227	
Age, years, mean ± SD	52.9 ± 14	
Sex, female:male (n)	729:498	
BMI, kg/m^2^, mean ± SD	23.7 ± 3.1	
ASA score, mean ± SD	1.8 (0.5)	
Tumor size, cm, mean ± SD	3.6 ± 2.3	
Diagnosis	n	**%**
Pancreatic cancer	218	17.8
Pancreatic ductal adenocarcinoma	196	
Intraductal papillary mucinous carcinoma	12	
Adenosquamous cancer	3	
Mixed acinar-neuroendocrine carcinoma	2	
Sarcomatoid cancer	2	
Anaplastic carcinoma	1	
Intraductal papillary mucinous neoplasm (IPMN)	238	19.4
IPMN low-grade dysplasia	122	
IPMN intermediate-grade dysplasia	99	
IPMN high-grade dysplasia	17	
Pancreatic neuroendocrine tumor (PNET)	122	9.9
PNET grade 1	64	
PNET grade 2	26	
PNET grade 3	7	
PNET unknown grade	25	
Solid pseudopapillary neoplasm	178	14.5
Mucinous cyst neoplasm	168	13.7
Serous cyst neoplasm	142	11.6
Pancreatitis (with or without pseudocyst)	78	6.4
Other pancreatic tumors	35	2.9

SD, standard deviation; BMI, body mass index; ASA, American Society of Anesthesiologists.

**Table 2:** Types of laparoscopic distal pancreatectomy.

Procedure	Patients (N = 1227)		
	n		%
LDPS	654		53.3
LDPS only	539		43.9
LDPS with combined operation	115		9.4
LDPS + Lap.SWR		19	
LDPS + Lap.SWR + Lap.CR		2	
LDPS + Lap.CR		11	
LDPS + Lap.CR + LC		2	
LDPS + Lap.CR + LC + Lap. ovarian resection		1	
LDPS + LC		67	
LDPS + Lap. ovarian resection		2	
LDPS + Lap. partial nephrectomy		1	
LDPS + Lap. portal vein resection		3	
LDPS + Lap. liver resection + LC		2	
LDPS + Lap. liver resection		1	
LDPS + Lap.SBR		4	
SP-LDP	573		46.7
SVP-SP-LDP	417		33.7
SVP-SP-LDP only	367		
SVP-SP-LDP with combined operation	50		
SVP-SP-LDP + LC		41	
SVP-SP-LDP +LDG		3	
SVP-SP-LDP + Lap-liver resection + LC		1	
SVP-SP-LDP + Lap-resection of hepatic cyst		2	
SVP-SP-LDP + Lap-ovarian resection		1	
SVP-SP-LDP + Lap-appendectomy		2	
SP-LDP with splenic vessels ligation (Lap-Warshaw)	156		12.6
Lap-Warshaw only	145		
Lap-Warshaw with combined operation	11		
Lap-Warshaw + LC		10	
Lap-Warshaw + Lap-ovarian resection		1	

LDPS, laparoscopic distal pancreatectomy with splenectomy; SP-LDP, spleen-preserving laparoscopic distal pancreatectomy; LC, laparoscopic cholecystectomy; Lap.CR, laparoscopic colectomy; Lap.SWR, laparoscopic stomach wedge resection; Lap.SBR, laparoscopic small-bowel resection.

**Table 3:** In-hospital complications in patients who underwent LDP.

Surgical Complications with Clavien Classification	N = 1227	%
No.	1065	86.8
Grade I	6	0.5
Chylous ascites with low long-chain triglyceride diet	3	
Superficial wound infection with bedside care	2	
Atelectasis	1	
Grade II	115	9.4
Pancreatic fistula or intra-abdominal fluid collection with antibiotic therapy	97	
Ileus	6	
Postoperative bleeding with transfusion	5	
Delayed gastric emptying	2	
Tractitis (drain insertion site)	1	
Portal vein thrombus with anticoagulant therapy	1	
Wound dehiscence	1	
Postoperative delirium	1	
Cerebral infarction with anticoagulant therapy	1	
Grade III	41	3.4
Grade IIIa	34	2.8
Pancreatic fistula grade B with intervention therapy	32	
EUS-guided gastrocystostomy	16	
Percutaneous drainage	11	
ERPD or ENPD insertion	5	
Intra-abdominal fluid collection with drainage	1	
Splenic artery aneurysmal bleeding with embolization	1	
Grade IIIb	7	0.6
Postoperative bleeding with reoperation	4	
Mechanical ileus with reoperation	2	
Common bile duct injury with reoperation	1	
2016 International Study Group Pancreatic Fistula		
No	681	55.5
Biochemical leak	488	39.8
Grade B	58	4.7

EUS, endoscopic ultrasonography; ERPD, endoscopic retrograde pancreatic drainage; ENPD, endoscopic nasopancreatic drainage.

**Table 4:** Logistic regression analysis of variables affecting postoperative complications in patients after laparoscopic distal pancreatectomy.

Factors	No. of Patients	Univariate (*p*)	Odds Ratio (95% CI)	Multivariate (*p*)
Overall complication				
Age (years)		0.715		
<70	1086 (88.5%)			
≥70	141 (11.5%)			
Sex		0.003		0.124
Female	729 (59.4%)			
Male	498 (40.6%)		1.317 (0.927–1.872)	
ASA score		0.516		
1	262 (21.4%)			
2	923 (75.2%)	0.827		
3	42 (3.4%)	0.260		
BMI		0.210		
Normal	799 (65.1%)			
Overweight	350 (28.5%)	0.165		
Obese	36 (2.9%)	0.200		
Preoperative albumin level		0.109		
<3.5 g/dL	163 (13.3%)			
≥3.5 g/dL	1064 (86.7%)			
Operative time		<0.001		< **0.001**
<200 min	691 (56.3%)			
≥200 min	536 (43.7%)		1.915 (1.341–2.735)	
Estimated blood loss		<0.001		**0.002**
<320 mL	1135 (92.5%)			
≥320 mL	92 (7.5%)		2.201 (1.322–3.664)	
Surgeon’s experience		<0.001		< **0.001**
Inexperienced	189 (15.4%)			
Experienced	1038 (84.6%)		0.442 (0.297–0.658)	
Splenectomy		<0.001		< **0.001**
Yes	654 (53.3%)			
No	573 (46.7%)		0.460 (0.314–0.673)	
Multivisceral resection		0.432		
No	1180 (96.2%)			
Yes	47 (3.8%)			
Length of resection		0.702		
<12 cm	979 (79.8%)			
≥12 cm	241 (19.6%)			
Malignancy		0.136		
Benign	978 (79.7%)			
Malignant	249 (20.3%)			
Major complication				
Age (years)		0.723		
<70	1086 (88.5%)			
≥70	141 (11.5%)			
Sex		0.009		**0.020**
Female	729 (59.4%)			
Male	498 (40.6%)		2.142 (1.125–4.079)	
ASA score		0.733		
1	262 (21.4%)			
2	923 (75.2%)	0.431		
3	42 (3.4%)	0.998		
BMI		0.752		
Normal	799 (65.1%)			
Overweight	350 (28.5%)	0.837		
Obese	36 (2.9%)	0.490		
Preoperative albumin level		0.102		
<3.5 g/dL	163 (13.3%)			
≥3.5 g/dL	1064 (86.7%)			
Operative time		0.002		**0.005**
<200 min	691 (56.3%)			
≥200 min	536 (43.7%)		2.648 (1.352–5.189)	
Estimated blood loss		0.023		0.230
<320 mL	1135 (92.5%)			
≥320 mL	92 (7.5%)		1.718 (0.711–4.154)	
Surgeon’s experience		0.015		**0.026**
Inexperienced	189 (15.4%)			
Experienced	1038 (84.6%)		0.453 (0.225–0.912)	
Splenectomy		0.026		0.337
Yes	654 (53.3%)			
No	573 (46.7%)		0.702 (0.341–1.446)	
Multivisceral resection		0.247		
No	1180 (96.2%)			
Yes	47 (3.8%)			
Length of resection		0.968		
<12 cm	979 (79.8%)			
≥12 cm	241 (19.6%)			
Malignancy		0.150		
Benign	978 (79.7%)			
Malignant	249 (20.3%)			

HR, hazard ratio; CI, confidence interval; BMI, body mass index; ASA, American Society of Anesthesiologists.

## References

[1] Mehrabi A., Hafezi M., Arvin J., Esmaeilzadeh M., Garoussi C., Emami G., Kössler-Ebs J., Müller-Stich B.P., Büchler M.W., Hackert T. (2015). A systematic review and meta-analysis of laparoscopic versus open distal pancreatectomy for benign and malignant lesions of the pancreas: It’s time to randomize. Surgery.

[2] Sulpice L., Farges O., Goutte N., Bendersky N., Dokmak S., Sauvanet A., Delpero J.R., Achbt French Pancreatectomy Study Group (2015). Laparoscopic distal pancreatectomy for pancreatic ductal adenocarcinoma: Time for a randomized controlled trial? Results of an all-inclusive national observational study. Ann. Surg..

[3] Shin S.H., Kim S., Song K.B., Hwang D.W., Lee J.H., Lee N., Lee J.W., Jun E., Park K.-M., Lee Y.-J. (2015). A Comparative Study of Laparoscopic vs Open Distal Pancreatectomy for Left-Sided Ductal Adenocarcinoma: A Propensity Score-Matched Analysis. J. Am. Coll. Surg..

[4] Song K.B., Kim S., Park J.B., Kim Y.H., Jung Y.S., Kim M.-H., Lee S.-K., Seo D.-W., Lee S.S., Park D.H. (2011). Single-center experience of laparoscopic left pancreatic resection in 359 consecutive patients: Changing the surgical paradigm of left pancreatic resection. Surg. Endosc..

[5] Mabrut J., Fernandezcruz L., Azagra J., Bassi C., Delvaux G., Weerts J., Fabre J., Boulez J., Baulieux J., Peix J. (2005). Laparoscopic pancreatic resection: Results of a multicenter European study of 127 patients. Surgery.

[6] McCullough T.C., Roth J.V., Ginsberg P.C., Harkaway R.C. (2004). Estimated Blood Loss Underestimates Calculated Blood Loss during Radical Retropubic Prostatectomy. Urol. Int..

[7] Dindo D., Demartines N., Clavien P.-A. (2004). Classification of Surgical Complications: A new proposal with evaluation in a cohort of 6336 patients and results of a survey. Ann. Surg..

[8] Bassi C., Marchegiani G., Dervenis C., Sarr M., Abu Hilal M., Adham M., Allen P., Andersson R., Asbun H.J., Besselink M.G. (2017). The 2016 update of the International Study Group (ISGPS) definition and grading of postoperative pancreatic fistula: 11 Years After. Surgery.

[9] Mazmudar A., Vitello D., Chapman M., Tomlinson J.S., Bentrem D.J. (2016). Gender as a risk factor for adverse intraoperative and postoperative outcomes of elective pancreatectomy. J. Surg. Oncol..

[10] Mendoza A.S., Han H., Ahn S., Yoon Y.-S., Cho J.Y., Choi Y. (2015). Predictive factors associated with postoperative pancreatic fistula after laparoscopic distal pancreatectomy: A 10-year single-institution experience. Surg. Endosc..

[11] Sell N.M., Pucci M.J., Gabale S., Leiby B.E., Rosato E.L., Winter J.M., Yeo C.J., Lavu H. (2015). The influence of transection site on the development of pancreatic fistula in patients undergoing distal pancreatectomy: A review of 294 consecutive cases. Surgery.

[12] Malleo G., Salvia R., Mascetta G., Esposito A., Landoni L., Casetti L., Maggino L., Bassi C., Butturini G. (2014). Assessment of a Complication Risk Score and Study of Complication Profile in Laparoscopic Distal Pancreatectomy. J. Gastrointest. Surg..

[13] Seeliger H., Christians S., Angele M.K., Kleespies A., Eichhorn M.E., Ischenko I., Boeck S., Heinemann V., Jauch K.-W., Bruns C.J. (2010). Risk factors for surgical complications in distal pancreatectomy. Am. J. Surg..

[14] Goh B.K., Tan Y.-M., Chung Y.-F.A., Cheow P.-C., Ong H.-S., Chan W.-H., Chow P.K., Soo K.-C., Wong W.-K., Ooi L.L.P.J. (2008). Critical Appraisal of 232 Consecutive Distal Pancreatectomies With Emphasis on Risk Factors, Outcome, and Management of the Postoperative Pancreatic Fistula. Arch. Surg..

[15] Sledzianowski J., Duffas J., Muscari F., Suc B., Fourtanier F. (2005). Risk factors for mortality and intra-abdominal morbidity after distal pancreatectomy. Surgery.

[16] Kleeff J., Diener M.K., Z’Graggen K., Hinz U., Wagner M., Bachmann J., Zehetner J., Muller M.W., Friess H., Buchler M.W. (2007). Distal Pancreatectomy: Risk factors for surgical failure in 302 consecutive cases. Ann. Surg..

[17] Dumitrascu T., Eftimie M., Aiordachioae A., Stroescu C., Dima S., Ionescu M., Popescu I. (2018). Male gender and increased body mass index independently predicts clinically relevant morbidity after spleen-preserving distal pancreatectomy. World J. Gastrointest. Surg..

[18] Tieftrunk E., Demir I.E., Schorn S., Sargut M., Scheufele F., Calavrezos L., Schirren R., Friess H., Ceyhan G.O. (2018). Pancreatic stump closure techniques and pancreatic fistula formation after distal pancreatectomy: Meta-analysis and single-center experience. PLoS ONE.

[19] Peng Y.-P., Zhu X.-L., Yin L.-D., Zhu Y., Wei J.-S., Wu J.-L., Miao Y. (2017). Risk factors of postoperative pancreatic fistula in patients after distal pancreatectomy: A systematic review and meta-analysis. Sci. Rep..

[20] Xia T., Zhou J.-Y., Mou Y.-P., Xu X.-W., Zhang R.-C., Zhou Y.-C., Chen R.-G., Lu C., Huang C.-J. (2017). Risk factors for postoperative pancreatic fistula after laparoscopic distal pancreatectomy using stapler closure technique from one single surgeon. PLoS ONE.

[21] Braga M., Pecorelli N., Ferrari D., Balzano G., Zuliani W., Castoldi R. (2014). Results of 100 consecutive laparoscopic distal pancreatectomies: Postoperative outcome, cost-benefit analysis, and quality of life assessment. Surg. Endosc..

[22] Kantor O., Bryan D.S., Talamonti M.S., Lutfi W., Winchester D.J., Prinz R.A., Sharpe S., Baker M.S. (2017). Laparoscopic Distal Pancreatectomy for Cancer Provides Oncologic Outcomes and Overall Survival Identical to Open Distal Pancreatectomy. J. Gastrointest. Surg..

[23] Ricci C., Casadei R., Taffurelli G., Toscano F., Pacilio C.A., Bogoni S., D’Ambra M., Pagano N., Di Marco M.C., Minni F. (2015). Laparoscopic Versus Open Distal Pancreatectomy for Ductal Adenocarcinoma: A Systematic Review and Meta-Analysis. J. Gastrointest. Surg..

[24] Riviere D., Gurusamy K.S., Kooby D.A., Vollmer C.M., Besselink M.G., Davidson B.R., Van Laarhoven C.J. (2016). Laparoscopic versus open distal pancreatectomy for pancreatic cancer. Cochrane Database Syst. Rev..

[25] Pannegeon V., Pessaux P., Sauvanet A., Vullierme M.-P., Kianmanesh R., Belghiti J. (2006). Pancreatic Fistula After Distal Pancreatectomy: Predictive risk factors and value of conservative treatment. Arch. Surg..

[26] Eshmuminov D., Schneider M.A., Tschuor C., A Raptis D., Kambakamba P., Muller X., Lesurtel M., Clavien P.-A. (2018). Systematic review and meta-analysis of postoperative pancreatic fistula rates using the updated 2016 International Study Group Pancreatic Fistula definition in patients undergoing pancreatic resection with soft and hard pancreatic texture. HPB.

[27] Cheng H., Clymer J.W., Chen B.P.-H., Sadeghirad B., Ferko N., Cameron C.G., Hinoul P. (2018). Prolonged operative duration is associated with complications: A systematic review and meta-analysis. J. Surg. Res..

[28] Fong Y., Gonen M., Rubin D., Radzyner M., Brennan M.F. (2005). Long-Term Survival Is Superior After Resection for Cancer in High-Volume Centers. Trans. Meet. Am. Surg. Assoc..

[29] McPhee J.T., Hill J.S., Whalen G.F., Zayaruzny M., Litwin D.E., Sullivan M.E., Anderson F.A., Tseng J.F. (2007). Perioperative Mortality for Pancreatectomy: A national perspective. Ann. Surg..

[30] Tang C.W., Feng W.M., Bao Y., Fei M.Y., Tao Y.L. (2014). Spleen-preserving Distal Pancreatectomy or Distal Pancreatectomy with Splenectomy?: Perioperative and patient-reported outcome analysis. J. Clin. Gastroenterol..

[31] Shoup M., Brennan M.F., McWhite K., Leung D.H.Y., Klimstra D., Conlon K.C. (2002). The value of splenic preservation with distal pancreatectomy. Arch. Surg..

[32] Pendola F., Gadde R., Ripat C., Sharma R., Picado O., Lobo L., Sleeman D., Livingstone A.S., Merchant N., Yakoub D. (2017). Distal pancreatectomy for benign and low grade malignant tumors: Short-term postoperative outcomes of spleen preservation-A systematic review and update meta-analysis. J. Surg. Oncol..

[33] Berkel A.E., Klaase J.M., De Graaff F., Brusse-Keizer M.G., Bongers B.C., Van Meeteren N.L. (2018). Patient’s Skeletal Muscle Radiation Attenuation and Sarcopenic Obesity are Associated with Postoperative Morbidity after Neoadjuvant Chemoradiation and Resection for Rectal Cancer. Dig. Surg..

[34] Gaujoux S., Cortès A., Couvelard A., Noullet S., Clavel L., Rebours V., Lévy P., Sauvanet A., Ruszniewski P., Belghiti J. (2010). Fatty pancreas and increased body mass index are risk factors of pancreatic fistula after pancreaticoduodenectomy. Surgery.

[35] Vanbrugghe C., Ronot M., Cauchy F., Hobeika C., Dokmak S., Aussilhou B., Ragot E., Gaujoux S., Soubrane O., Lévy P. (2018). Visceral Obesity and Open Passive Drainage Increase the Risk of Pancreatic Fistula Following Distal Pancreatectomy. J. Gastrointest. Surg..

[36] Zhai T.-S., Zhang B., Qu Z., Chen C. (2018). Elevated visceral obesity quantified by CT is associated with adverse postoperative outcome of laparoscopic radical nephrectomy for renal clear cell carcinoma patients. Int. Urol. Nephrol..

[37] Ri M., Aikou S., Seto Y. (2017). Obesity as a surgical risk factor. Ann. Gastroenterol. Surg..

[38] Lee S.E., Jang J.-Y., Lim C.-S., Kang M.J., Kim S.H., Kim M.-A., Kim S.-W. (2010). Measurement of Pancreatic Fat by Magnetic Resonance Imaging: Predicting the occurrence of pancreatic fistula after pancreatoduodenectomy. Ann. Surg..

[39] Karastergiou K., Smith S.R., Greenberg A.S., Fried S.K. (2012). Sex differences in human adipose tissues—The biology of pear shape. Boil. Sex Differ..

[40] Kollár D., Húszár T., Pohárnok Z., Cselovszky É., Oláh A. (2016). A Review of Techniques for Closure of the Pancreatic Remnant following Distal Pancreatectomy. Dig. Surg..

[41] Okano K., Kakinoki K., Suto H., Oshima M., Maeda N., Kashiwagi H., Yamamoto N., Akamoto S., Fujiwara M., Takama H. (2011). Slow parenchymal flattening technique for distal pancreatectomy using an endopath stapler: Simple and safe technical management. Hepatogastroenterology.

[42] Eguchi H., Nagano H., Tanemura M., Takeda Y., Marubashi S., Kobayashi S., Wada H., Umeshita K., Mori M., Doki Y. (2011). A Thick Pancreas Is a Risk Factor for Pancreatic Fistula after a Distal Pancreatectomy: Selection of the Closure Technique according to the Thickness. Dig. Surg..

[43] Adachi T., Kuroki T., Kitasato A., Hirabaru M., Matsushima H., Soyama A., Hidaka M., Takatsuki M., Eguchi S. (2015). Safety and efficacy of early drain removal and triple-drug therapy to prevent pancreatic fistula after distal pancreatectomy. Pancreatology.

[44] Bassi C., Molinari E., Malleo G., Crippa S., Butturini G., Salvia R., Talamini G., Pederzoli P. (2010). Early Versus Late Drain Removal After Standard Pancreatic Resections. Ann. Surg..

[45] Kawai M., Tani M., Terasawa H., Ina S., Hirono S., Nishioka R., Miyazawa M., Uchiyama K., Yamaue H. (2006). Early Removal of Prophylactic Drains Reduces the Risk of Intra-abdominal Infections in Patients With Pancreatic Head Resection. Ann. Surg..

